# Autologous Tenon's capsule for post-vitrectomy macular hole closure: A case report

**DOI:** 10.1016/j.ajoc.2026.102635

**Published:** 2026-07-29

**Authors:** Maryam Al-Muzaffar, Anant Pai, Osman Abdelzaher, Hashem Abu Serhan

**Affiliations:** a College of Medicine, Qatar University, Doha, Qatar; b Department of Ophthalmology, Hamad Medical Corporation, Doha, Qatar

**Keywords:** Macular hole, Tenon's capsule graft, Pars plana vitrectomy, Retinal detachment, Retina

## Abstract

**Purpose:**

To report the anatomical and visual outcomes of autologous Tenon's capsule patch grafting for closure of a large post-vitrectomy full-thickness macular hole.

**Observations:**

A 63-year-old female with proliferative diabetic retinopathy presented with decreased vision in the right eye. Optical coherence tomography demonstrated a 1112 μm full-thickness macular hole. Due to prior internal limiting membrane peeling, insufficient tissue remained for conventional techniques, an autologous Tenon's capsule graft was harvested through a limited conjunctival peritomy and positioned on the macular defect. Silicone oil tamponade was used to stabilize the graft. One-month follow-up demonstrated anatomical closure of the macular hole, which remained stable at 6 months. Visual acuity improved from hand motion preoperatively to 1/60 at final follow-up.

**Conclusions and importance:**

Autologous Tenon's capsule patch grafting may serve as a useful surgical alternative for large post-vitrectomy macular holes when internal limiting membrane tissue is unavailable.

## 1 Introduction

Macular holes (MHs) are an anatomical defect that leads to an interruption of the neurosensory retinal layer from the internal limiting membrane (ILM) to the retinal pigment epithelium (RPE) at the center of the fovea, leading to an appreciable central vision loss.^1^^,^^2^

Primary (idiopathic) MHs are a result of traction on the retina caused by an anomalous posterior vitreous detachment (PVD). On the other hand, secondary MHs occur as a result of a pathologic mechanism such as surgery, trauma, or inflammation and require a careful treatment approach.^3^^,^^4^

Currently, the management of MHs is pars plana vitrectomy (PPV), removal of the posterior hyaloid and epiretinal membranes, ILM peeling, and gas tamponade.^1^^,^^2^^,^^5^

Despite the MHs closure rate of 85-90% after the primary surgery, the rate of persistence is between 8% and 44%.^2^ This can be attributed to the stage of MH, its size, chronicity, patient nonadherence with maintaining a prone posture in the postoperative phase, and residual epiretinal membranes (ERMs).^1^ Moreover, restoring foveal integrity can be challenging when dealing with secondary or persistent defects along with an insufficient or unavailable ILM.^2^ Recent advances explore biologic tissue grafts, including autologous lens capsule, amniotic membrane, and, more recently, Tenon's capsule patch graft (TCPG), as alternatives when facing such a challenge.^4^ Tenon's grafts provide a biocompatible collagenous substrate that facilitates cellular proliferation and photoreceptor alignment across the hole, while eliminating the need for exogenous material.^4^

Herein, we describe a case where an autologous TCPG was used to close a 990 μm full-thickness MHs in a patient with proliferative diabetic retinopathy who had previously undergone pars plana vitrectomy with removal of epiretinal membrane along with ILM. This approach demonstrated favorable anatomical and visual outcomes, highlighting the evolving role of Tenon's tissue as a viable surgical alternative in managing such cases.

## 2 Case presentation

A 63-year-old female presented with a history of poor vision of one month's duration. She had a past medical history of type 2 diabetes mellitus complicated by macroalbuminuric nephropathy and hypertension. Her ocular history was significant for bilateral cataract surgery, and bilateral center involving diabetic macular edema with high-risk proliferative diabetic retinopathy (PDR). Patient had also received five intravitreal ranibizumab injections and panretinal photocoagulation (PRP) in both eyes. The left eye had remained stable with the BCVA of 6/9 following PRP and anti-VEGF therapy, with no prior vitrectomy or macular surgery. The patient gave a history of undergoing pars plana vitrectomy for epiretinal membrane in the right eye few weeks prior to presentation. There was no history of ocular trauma, uveitis, or other intraocular procedures.

On examination, the best-corrected visual acuity (BCVA) was hand motion in the right eye Intraocular pressure (IOP) was within 14 mmHg. The anterior segment showed a clear cornea, a well-centered posterior chamber intraocular lens, and an open posterior capsule.

**Fig. 1:**
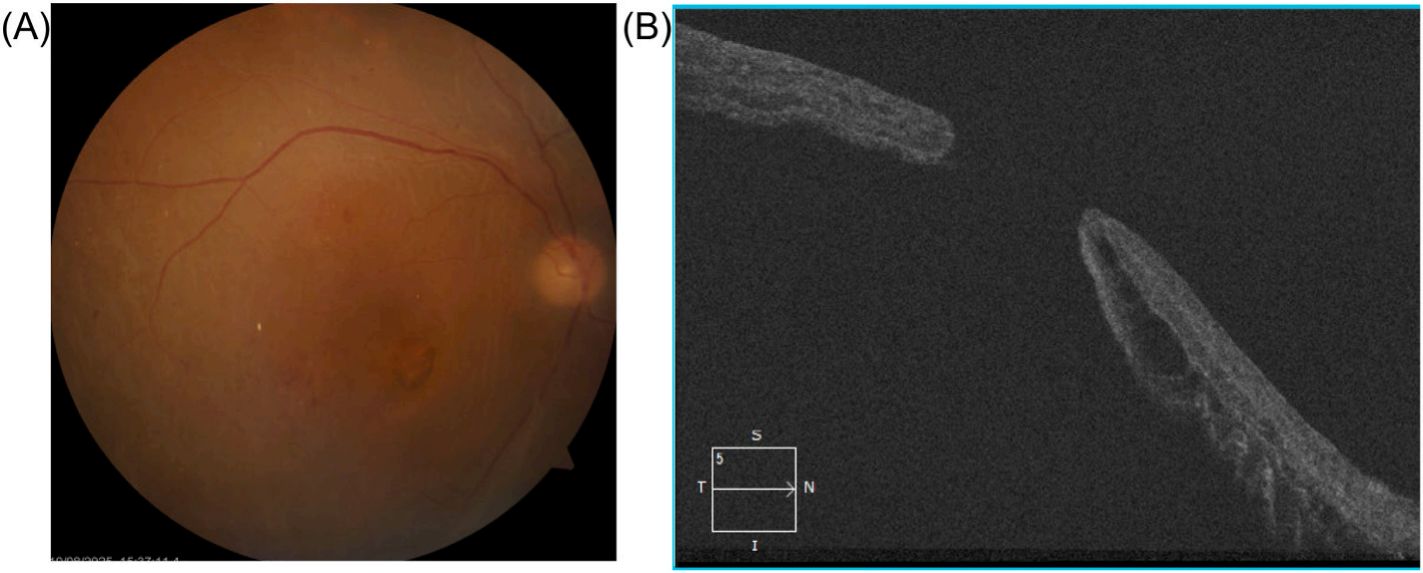
(A) Fundus photograph of the right eye demonstrating a full-thickness macular hole with surrounding retinal changes and prior panretinal photocoagulation scars. (B) Optical coherence tomography (OCT) demonstrating a large full-thickness macular hole with surrounding intraretinal cystic changes. The apparent inferior displacement of the foveal center on fundus photography is attributable to the large amount of subretinal fluid present.

Fundus examination of the right eye revealed a regressed PDR with attached retina from temporal vascular arcades to the periphery and evidence of prior panretinal photocoagulation (PRP) scars. However, the retina was detached in the posterior pole between the temporal vascular arcades and a 1112 μm full-thickness macular hole with adjacent cystoid changes (Fig. 1A and B).

## 3 Surgical technique

Given the large size of the macular defect, we planned for resurgery with ILM graft. Intraoperatively, we found that the ILM was absent over a large area around the macular hole till inferior and superior vascular arcades. Given the absence of sufficient residual ILM tissue, lasered attached peripheral retina, absence of posterior capsule and non-availability of amniotic membrane, an alternative scaffold technique using an autologous Tenon's capsule graft was planned intra-operatively. The graft was harvested from the anterior Tenon's capsule through a limited conjunctival peritomy, trimmed approximately 1 × 1 mm matching the hole size, and carefully positioned on the macular defect. Subretinal fluid was drained through a pre-existing peripheral retinal break prior to graft placement. Silicone oil tamponade was used to stabilize the graft and support anatomical closure.

## 4 Follow up

**Fig. 2:**
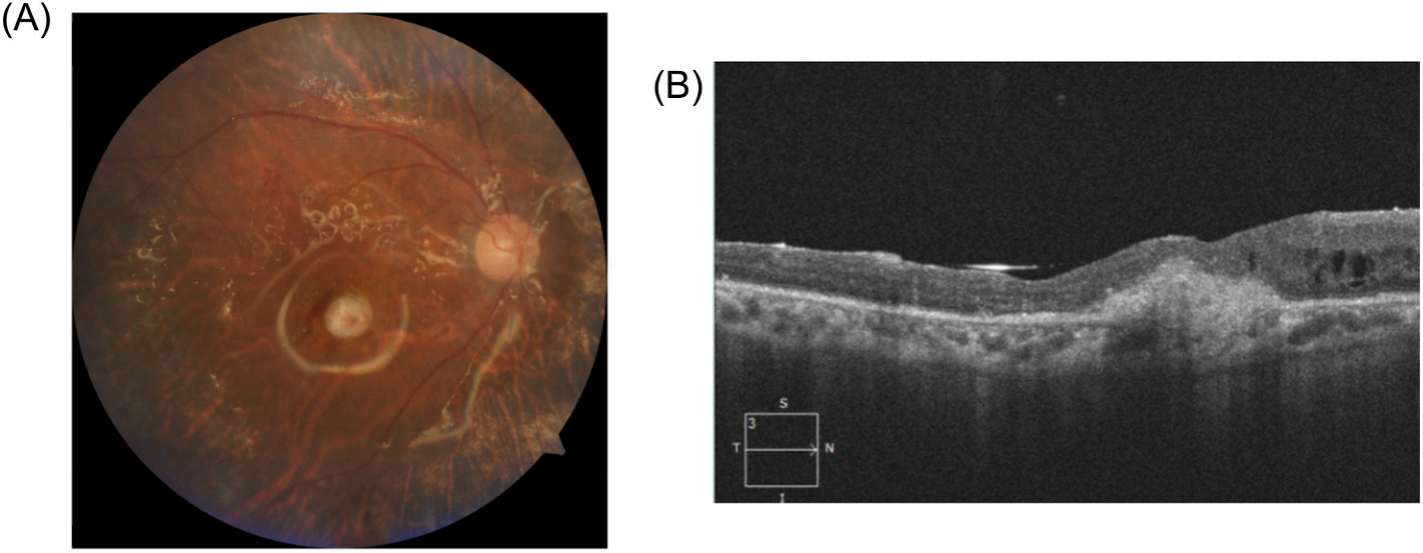
(A) Fundus photograph showing postoperative macular appearance one month after autologous Tenon's capsule patch graft surgery. (B) Optical coherence tomography obtained at 1-month follow-up demonstrating anatomical closure of the macular hole.

At 1-month follow-up, visual acuity improved from HM preoperatively to 1/60. Fundus examination demonstrated a stable postoperative macular appearance (Fig. 2A), while OCT showed anatomical closure of the macular hole (Fig. 2B).

**Fig. 3:**
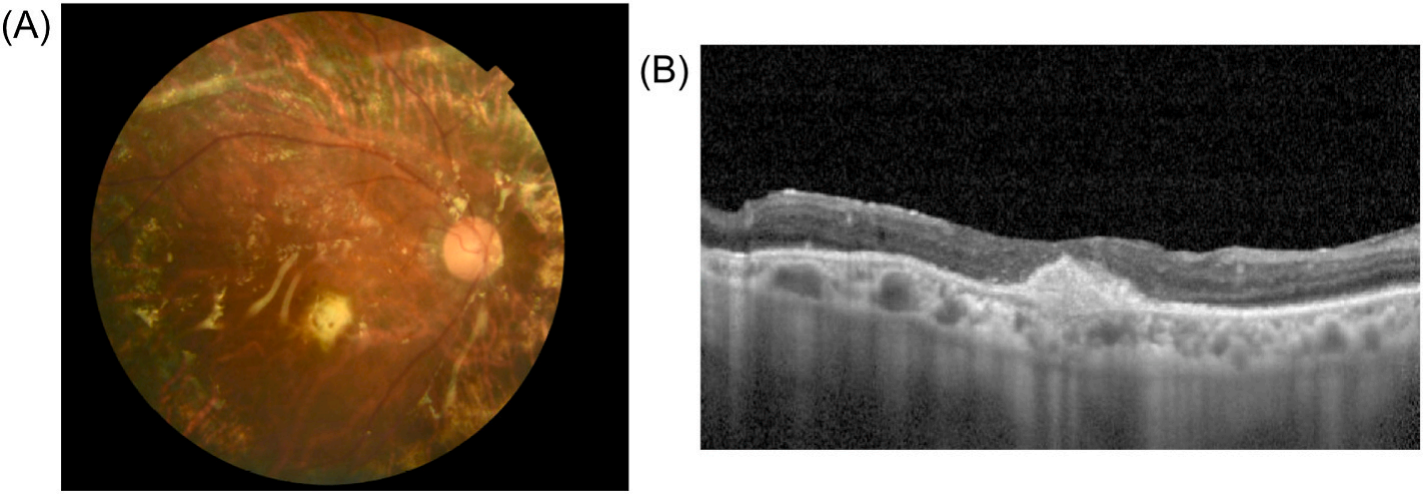
(A) Fundus photograph showing stable postoperative macular appearance at follow-up. (B) Optical coherence tomography obtained at 6-month follow-up demonstrating maintained anatomical closure of the macular hole.

At 6-month follow-up, fundus examination demonstrated stable postoperative macular appearance (Fig. 3A). Interestingly, OCT showed a maintained anatomical closure of the macular hole with macular anatomical reattachment. Subretinal scar-like material was noted at the graft-retina interface (Fig. 3B). The retina remained stable during follow-up with no intraoperative or postoperative complications.

## 5 Discussion

Large post-vitrectomy MHs represent a challenging surgical scenario, particularly when ILM tissue has already been removed and conventional techniques cannot be performed.^2^ In such cases, alternative scaffold-based approaches may facilitate retinal defect closure. Several biological grafts have been described for complex MHs, including autologous retinal grafts, lens capsule grafts, and amniotic membrane transplantation.^4^ Recent case reports have also described the successful use of autologous Tenon's capsule grafts in the management of complex or persistent macular holes, particularly in eyes with prior internal limiting membrane peeling.^3^^,^^6^^,^^7^ These reports demonstrate favorable anatomical closure rates, supporting the role of Tenon's tissue as an effective biological scaffold in such challenging cases. To our knowledge, the use of TCPG specifically for a secondary full-thickness macular hole arising as a complication of vitrectomy has not been previously reported.

The use of the Tenon's capsule as a biologic scaffold to repair MHs is supported by its distinctive histological and structural properties.^8^^,^^9^ The capsule consists of a dense, elastic, and vascular fibrous connective tissue encircling the globe, forming a smooth interface between the sclera and the orbital fat.^8^^,^^9^ The Tenon's capsule is composed of two components: a thick fibrous tissue with smooth muscle fibers anteriorly and a posterior fine fibrous capsule, which merges with the orbital fat and fuses with the meninges around the optic nerve.^10^^,^^11^ This unique architecture gives it a mechanical strength and pliability, facilitating its integration with subretinal or macular space.^8^ Another feature that favors the usage of the Tenon's capsule is that its average thickness is 234μm making it thicker than an ILM or an amniotic membrane which allows for smoother manipulation.^10^ Adding to this, an autologously harvested Tenon's capsule serves as a biocompatible collagen matrix promoting Müller cell migration and gliotic bridging across the foveal defect, along with its natural elasticity and vascular potential, can support nutrient diffusion and cellular proliferation at the graft-retina interface.^12^

In our case, using the Tenon's capsule to fill the MHs resulted in an anatomical closure and visual improvement. However, it is worth noting that the modest visual improvement observed may be attributed to the anatomical reattachment of the macula rather than the MH closure. This is supported by evidence that using a biologic material can contribute to retinal repair by providing both mechanical scaffolding and a favorable microenvironment.

Niffenegger et al. emphasized that inflammation and retinal edema contribute to persistent MHs demonstrating that restoring retinal homeostasis can lead to closure even without surgical intervention.^12^ The Tenon's graft likely contributed not only as a physical filler but also by stabilizing the microenvironment, facilitating glial proliferation, and promoting anatomical reattachment.^12^

Tenon's capsule offers some advantages compared to other alternatives like amniotic membrane or lens capsule grafts. Tenon's capsule is autologous, inexpensive, readily available, and histologically compatible.^9^ Additionally, it can be harvested using a small peritomy, which is straightforward and does not require any special tissue handling techniques.

This case carries a notable limitation. Silicone oil removal was offered to the patient at 2 months postoperatively; however, the patient repeatedly postponed it and was subsequently lost to follow-up after 6 months. Therefore, the long term anatomical and functional outcomes following the silicone oil removal remain unknown.

## 6 Conclusion

Overall, this case supports the potential use of TCPG as an option for the closure of MHs due to its complex anatomical and biological components that further contribute to retinal healing. Although this option represents a safe and interesting approach with satisfactory early results, further studies are needed to evaluate the long-term outcomes better and to standardize a surgical technique and indications.

## Patient consent

Written informed consent was obtained from the patient for publication of this case report and accompanying images.

## Authorship

All authors attest that they meet the current ICMJE criteria for Authorship.

## Funding

No funding or grant support

## CRediT authorship contribution statement

**Maryam Al-Muzaffar:** Conceptualization, Data curation, Funding acquisition, Investigation, Validation, Visualization, Writing – original draft, Writing – review & editing. **Anant Pai:** Conceptualization, Data curation, Supervision, Validation, Visualization, Writing – original draft, Writing – review & editing. **Osman Abdelzaher:** Methodology, Project administration, Resources, Software, Writing – review & editing. **Hashem Abu Serhan:** Conceptualization, Supervision, Validation, Visualization, Writing – original draft, Writing – review & editing.

## Declaration of competing interest

The authors declare that they have no known competing financial interests or personal relationships that could have appeared to influence the work reported in this paper.

## Figures and Tables

**Supplementary Fig. 1:**
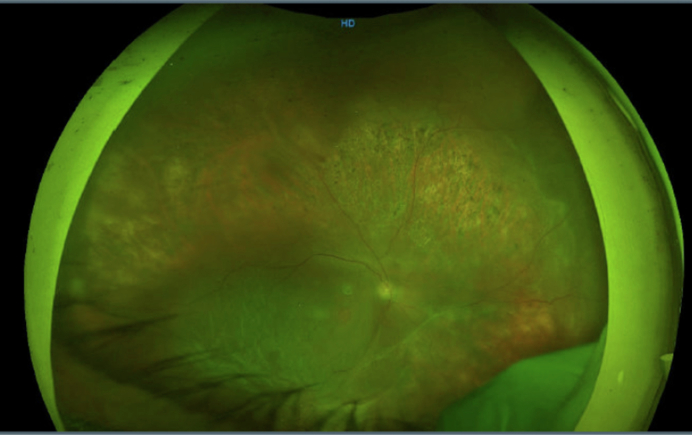
Wide-field fundus photograph of the right eye demonstrating the extent of panretinal photocoagulation and the posterior pole at presentation.
